# Harnessing engineered mesenchymal stem cell-derived extracellular vesicles for innovative cancer treatments

**DOI:** 10.1186/s13287-025-04708-5

**Published:** 2025-11-18

**Authors:** Wei-Lun Hwang, Shiu-Wen Huang, An-Ching Hsiao, Chin-Yau Chen, Ke-Fong Hsu, Yi-Tsen Hsieh, Tsai-Tsen Liao

**Affiliations:** 1 Department of Biotechnology and Laboratory Science in Medicine, National Yang Ming Chiao Tung University, Taipei, 11221 Taiwan; 2 Cancer and Immunology Research Center, National Yang Ming Chiao Tung University, Taipei, 11221 Taiwan; 3 Graduate Institute of Medical Sciences, College of Medicine, Taipei Medical University, 250 Wu-Hsing Street, Taipei, 11031 Taiwan; 4 Department of Pharmacology, School of Medicine, College of Medicine, Taipei Medical University, Taipei, Taiwan; 5 Translational Imaging Research Center & Research Center of Thoracic Medicine and Asthma, Taipei Medical University Hospital, Taipei, Taiwan; 6 Department of Surgery, National Yang Ming Chiao Tung University Hospital, Yilan, Taiwan; 7 Department of Medical Education, Cathay General Hospital, Taipei 106, Taiwan; 8 Education Center, College of Medicine, National Cheng Kung University Hospital, National Cheng Kung University, Tainan, 704 Taiwan; 9 Research Center of Cancer Translational Medicine, Taipei Medical University, Taipei, 110 Taiwan; 10 Cell Physiology and Molecular Image Research Center, Wan Fang Hospital, Taipei Medical University, New Taipei City, 235 Taiwan; 11 Cancer Research Center, Taipei Medical University Hospital, Taipei, Taiwan

**Keywords:** Mesenchymal stem/Stromal cells (MSCs), Extracellular vesicles (EVs), Bioengineering, Cancer therapy, RNA delivery

## Abstract

Mesenchymal stem/stromal cells (MSCs) are known for their regenerative and immunomodulatory capabilities, which have made them the focus of extensive therapeutic research. A growing body of evidence underscores that mesenchymal stem cell-derived extracellular vesicles (MSC-EVs) have emerged as a promising cell-free platform that mimics the therapeutic benefits of MSCs while mitigating their associated risks. This review synthesizes recent advancements in bioengineering strategies aimed at enhancing the therapeutic efficacy, targeting specificity, and cargo-loading capacity of MSC-EVs for cancer treatment. These strategies include endogenous and exogenous modification approaches. Endogenous strategies involve genetically modifying parental MSCs or using environmental preconditioning to modulate the extracellular vesicles (EVs) content or surface proteins of EVs they produce. Exogenous techniques include post-isolation loading of therapeutic cargo, such as small interfering RNAs (siRNAs) and microRNAs (miRNAs), as well as EV membrane modifications. We also highlight key preclinical and clinical findings, addressing the dual role of MSC-EVs, which can be either pro- or anti-tumorigenic depending on the MSC tissue origin and the tumor microenvironment. Notably, EVs derived from human umbilical cord-derived mesenchymal stem cells (hUC-MSCs) show the most consistent tumor-suppressive activity, making them a preferred choice for clinical development. Despite challenges related to production scalability, cargo-loading efficiency, and regulatory standardization, engineered MSC-EVs are poised to become a transformative platform in next-generation, cell-free precision cancer therapies. Future efforts, including the establishment of protocols compliant with Good Manufacturing Practice (GMP) and the integration of engineering advancements, will be essential for advancing healthcare innovations.

**Supplementary Information:**

The online version contains supplementary material available at 10.1186/s13287-025-04708-5.

Graduate Institute of Medical Sciences, College of Medicine, Taipei Medical University, Taipei, Taiwan, 250 Wu-Hsing Street, Taipei 11,031, Taiwan. E-mail: liaotsaitsen@tmu.edu.tw, ORCID: https://orcid.org/0000-0001-9185-9926; Tel.: 886-27361661 ext. 3435.

## Background

Mesenchymal stem/stromal cells (MSCs) exhibit multidirectional differentiation potential and can differentiate in vitro into multiple lineages, including osteoblasts, chondrocytes, and adipocytes [1]. In addition to their differentiation potential, MSCs exhibit strong immunomodulatory and regenerative properties, positioning them as valuable candidates for therapeutic applications. Notably, MSCs can actively migrate to sites of inflammation and modulate immune responses [2, 3]. Given that the tumor microenvironment (TME) exhibits features similar to those of chronic inflammation, it serves as a comparable target for MSC homing. Increasing attention has been given to their therapeutic potential in oncology. The ability of MSCs to home to tumor sites and integrate into the tumor stroma enables their use as a targeted delivery platform for anticancer therapies [4]. To exploit this tumor-tropic property, MSCs have been engineered to deliver therapeutic proteins, microRNAs (miRNAs), prodrugs, chemotherapy drugs, and oncolytic viruses [5, 6]. These strategies are designed to induce tumor cell apoptosis or activate immune responses that suppress tumor growth. However, despite these advantages, some studies suggest that MSCs may exert dual effects on tumor biology. The tumor-promoting effects of MSCs have been demonstrated in various cancers, including colorectal cancer (CRC), head and neck squamous cell carcinoma (HNSCC), gastric cancer, chronic lymphoid leukemia, and ovarian cancers [7–12]. These effects are largely attributed to the capacity of MSCs to modulate immune responses in an immunosuppressive environment, induce epithelial‒mesenchymal transition (EMT), promote drug resistance, and facilitate the expansion or reprogramming of cancer stem cells [13]. Therefore, the use of unmodified MSCs in cancer therapy carries potential oncogenic risks that warrant careful consideration. To mitigate these concerns and enhance therapeutic efficacy, researchers are increasingly focusing on genetically modified MSCs and their secreted extracellular vesicles (EVs) as targeted delivery systems for anticancer molecules.

Recently, mesenchymal stem cell-derived extracellular vesicles (MSC-EVs) have gained attention because of their role in intercellular communication and their ability to replicate many of the therapeutic benefits of parent MSCs without the safety risks associated with live cell therapies, such as tumorigenicity or immune rejection [14, 15]. Furthermore, MSC-EVs retain the tumor-homing capacity of MSCs, making them attractive candidates for targeted cancer therapy. EVs are lipid bilayer particles derived from cell membranes that function as natural carriers of molecular cargo. They transport a variety of bioactive molecules, such as nucleic acids, including deoxyribonucleic acid (DNA), ribonucleic acid (RNA), messenger RNA (mRNA), and miRNA, as well as proteins, lipids, and metabolites, to recipient cells and subsequently modulate cellular biological processes [16]. The engineering of MSC-EVs to deliver exogenous therapeutic molecules represents a promising new strategy in cancer therapy. This review summarizes recent progress in the field and examines the molecular mechanisms through which engineered MSC-EVs contribute to cancer therapy.

## Definition and classification of EVs

EVs were first separated and recorded by Peter Wolf via electron microscopy images and described as ‘a material in minute particulate form, sedimentable by high-speed centrifugation, and originating from platelets, but distinguishable from intact platelets’ in 1967 [17]. EVs are membrane-derived lipid-bound vesicles released by cells into the extracellular space to facilitate cell-to-cell communication and modulate the biological processes of cells. On the basis of the guidelines of Minimal Information for Studies of Extracellular Vesicles 2018 (MISEV2018) by the International Society for Extracellular Vesicles (ISEV), the generic term “EV” will be suggested for use in the nomenclature of all vesicles naturally released from cells, delimited by a lipid bilayer, and incapable of replication owing to the absence of a functional nucleus [18]. The update Minimal Information for Studies of Extracellular Vesicles 2023 (MISEV2023) redefines EVs by eliminating the requirement that EVs be ‘naturally released’, thus encompassing EVs derived under engineered or induced conditions [19, 20].

Previously, EVs were typically divided into three primary categories based on their origin: exosomes, ectosomes, and apoptotic bodies (ApoBDs) [21–23]. Despite differences in their biogenesis, these vesicle types frequently overlap in size and molecular composition, making their distinction in practical settings technically challenging. Reflecting these limitations, the MISEV2023 guidelines advocate the use of operational terms that describe EVs on the basis of measurable characteristics such as size, density, molecular composition, and cellular origin. For example, EVs can be categorized by size into small extracellular vesicles (sEVs, typically < 200 nm) and medium/large extracellular vesicles (m/lEVs, typically >200 nm) or by density into low-, medium-, and high-density vesicles. In addition to naturally occurring EVs, MISEV2023 also recognizes synthetic or engineered vesicles, including EV mimetics, artificial cell-derived vesicles (ACDVs), and synthetic vesicles (SVs), as part of the broader EV research landscape. The updated EV nomenclature guidelines are summarized in Table 1 [19]. These refined definitions and classifications aim to promote standardization, reproducibility, and clarity in EV-related research, especially in translational and therapeutic studies.

**Table 1:**
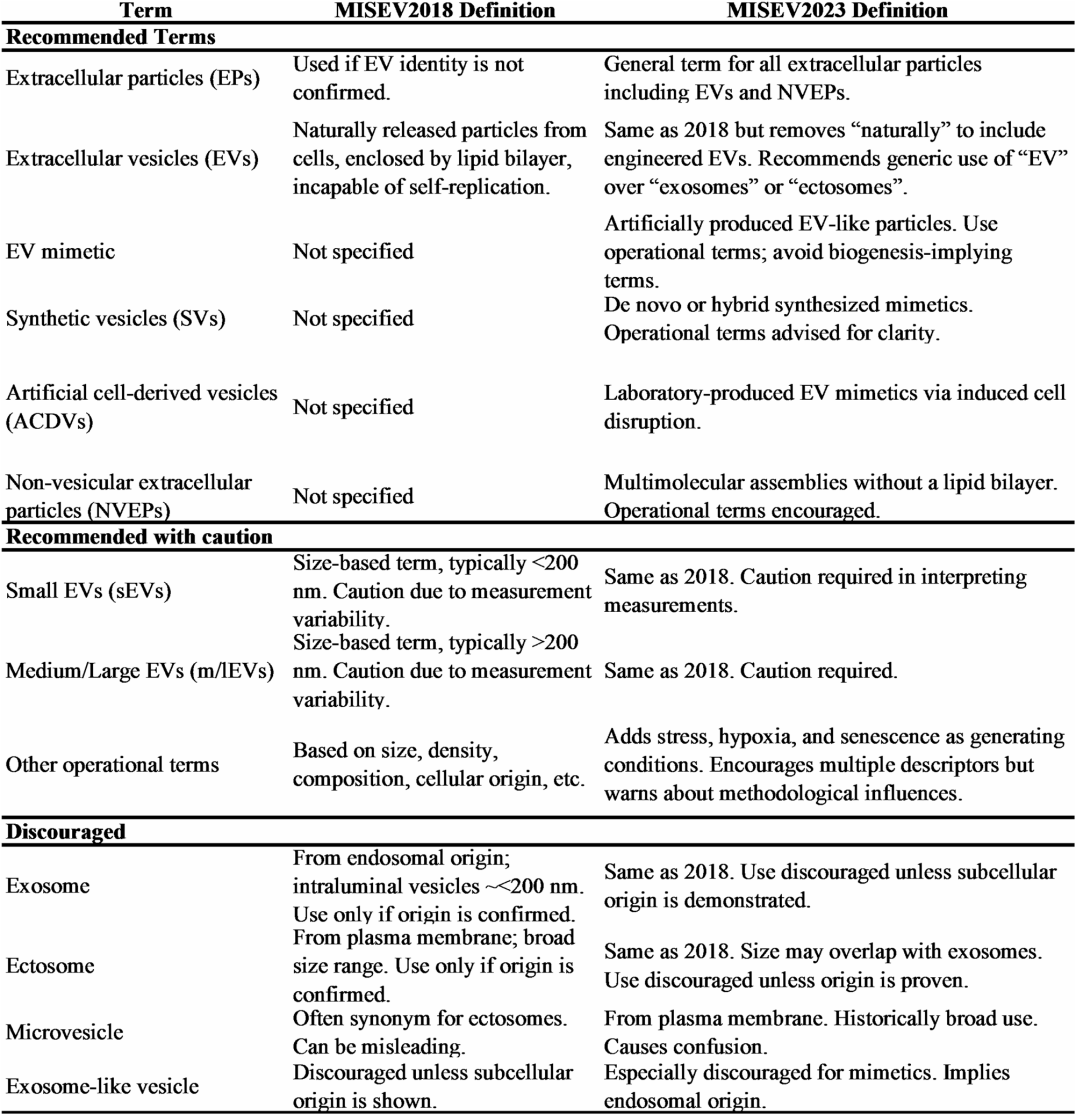
Classification and nomenclature guidelines for EVs

## Therapeutic potential of MSC-EVs in cancer

EVs are emerging as promising alternatives to traditional delivery systems like lipid nanoparticles (LNPs) for RNA-based cancer therapies. Although LNPs are widely studied in both preclinical and clinical settings, a significant hurdle is their inefficient endosomal escape, which is estimated at less than 2% for small interfering RNAs (siRNAs), successfully reaching the cytosol after endocytosis [24]. Furthermore, Maugeri et al. provided insight into the fate of the remaining LNP material, showing that much of it is incorporated into endocytosis-derived extracellular vesicles (endo-EVs). These endo-EVs, which are secreted following the cellular uptake of LNP-mRNA, are capable of protecting and delivering exogenous RNA in vivo. In mouse models, they have been shown to facilitate human protein production, with detectable levels found in plasma and various organs. Furthermore, compared with LNPs, endo-EVs induce significantly lower expression of inflammatory cytokines, making them a potentially safer alternative for RNA delivery [25]. These findings highlight the potential of harnessing engineered MSC-EVs as safe and efficient platforms for RNA-based therapies.

On the other hand, although full-length mRNAs can be loaded into EVs, this process leads to an increase in EV size and a decrease in the number of EVs, which presents challenges in scalability in clinical or commercial applications [26, 27]. In contrast, small RNA molecules—such as siRNAs and particularly miRNAs—are preferentially encapsulated within EVs, suggesting that an endogenous loading system exists within cells. This selective loading is regulated by specific cellular mechanisms. RNA-binding proteins such as heterogeneous nuclear ribonucleoprotein A2/B1 (hnRNPA2B1), Argonaute-2(AGO2), and ALG-2-interacting protein X(Alix) recognize conserved sequence motifs within miRNAs, mediating their packaging into EVs [28–30]. Mutation of these motifs disrupts miRNA loading, highlighting the presence of an active and selective cellular sorting system. As a result, certain miRNAs are enriched in EVs relative to their abundance in parent cells, whereas other RNA species are less efficiently incorporated. This selective enrichment has profound implications for intercellular communication, disease progression, and the development of EV-based diagnostic and therapeutic strategies.

Cell-derived EVs have opened new horizons in modern therapy for advanced drug delivery and therapeutic applications because of their key features, such as low immunogenicity, high physicochemical stability, capacity to penetrate tissues, and innate capacity to communicate with other cells over long distances. In particular, the functional effects of EVs on recipient cells are largely dictated by their molecular composition and surface proteins, which vary depending on the origin of the source cells [31]. Among various cellular sources, MSCs are considered particularly promising sources of EVs for clinical application because of their ability to be isolated from various tissues and their high capacity for ex vivo expansion [32, 33]. Furthermore, MSC-derived EVs have demonstrated the intrinsic tumor-homing capability of parent cells, making them well suited as delivery vehicles for targeted cancer therapy [34]. To harness MSC-EVs as carriers in cancer therapy, the genetic and pharmacological engineering of MSCs has been proposed as a viable strategy for delivering effective antitumor medications, such as chemotherapeutic compounds, miRNAs, and siRNAs, preferentially to cancer cells.

## Engineering strategies to enhance MSC-EV functionality

**Fig. 1:**
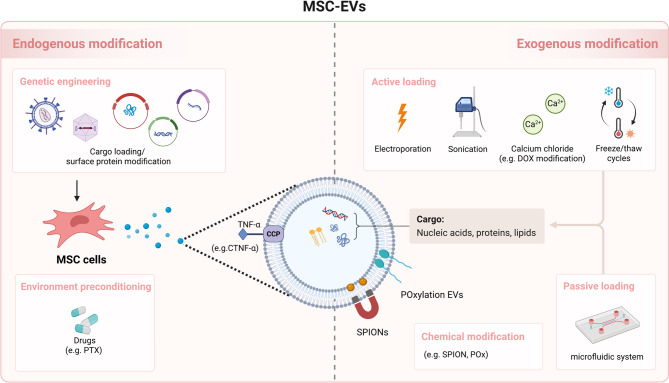
Representative strategies for the engineering of MSC-EVs. MSC-EVs can be engineered via two principal approaches: endogenous modification, which involves genetic manipulation or environmental preconditioning of parental MSCs prior to EV isolation, and exogenous modification, which involves direct manipulation of isolated EVs. Endogenous strategies include genetic engineering for therapeutic cargo enrichment or surface protein display (e.g., fusion protein cell penetrating peptide with tumor necrosis factor-alpha [CTNF-α fusion protein]), as well as preconditioning with therapeutic drugs to alter the composition. Exogenous strategies are classified into two categories: active loading, which includes electroporation, sonication, calcium chloride treatment, and freeze–thaw cycles for the incorporation of nucleic acids, proteins, or small molecules; and passive loading, such as microfluidic-assisted encapsulation of hydrophobic compounds. Additionally, surface chemical modifications (e.g., conjugation with superparamagnetic iron oxide nanoparticles [SPIONs] or polyoxazolines [POx]) can be applied to increase the efficiency, stability, and pharmacokinetic properties. The figure was created in BioRender

The application of MSC-EVs for the delivery of anticancer drugs, noncoding RNAs, and proteins presents a promising avenue for cancer therapy. Advanced bioengineering techniques, such as surface modification, targeted ligand incorporation, and cargo loading optimization, can be employed to improve the specificity, stability, and overall efficacy of MSC-EV-based therapeutic systems (Fig. 1). The engineering of MSC-EVs for drug loading and functional modification can be strategically implemented via two primary approaches (Table 2): (1) endogenous modification and (2) exogenous modification [35, 36].

**Table 2:**
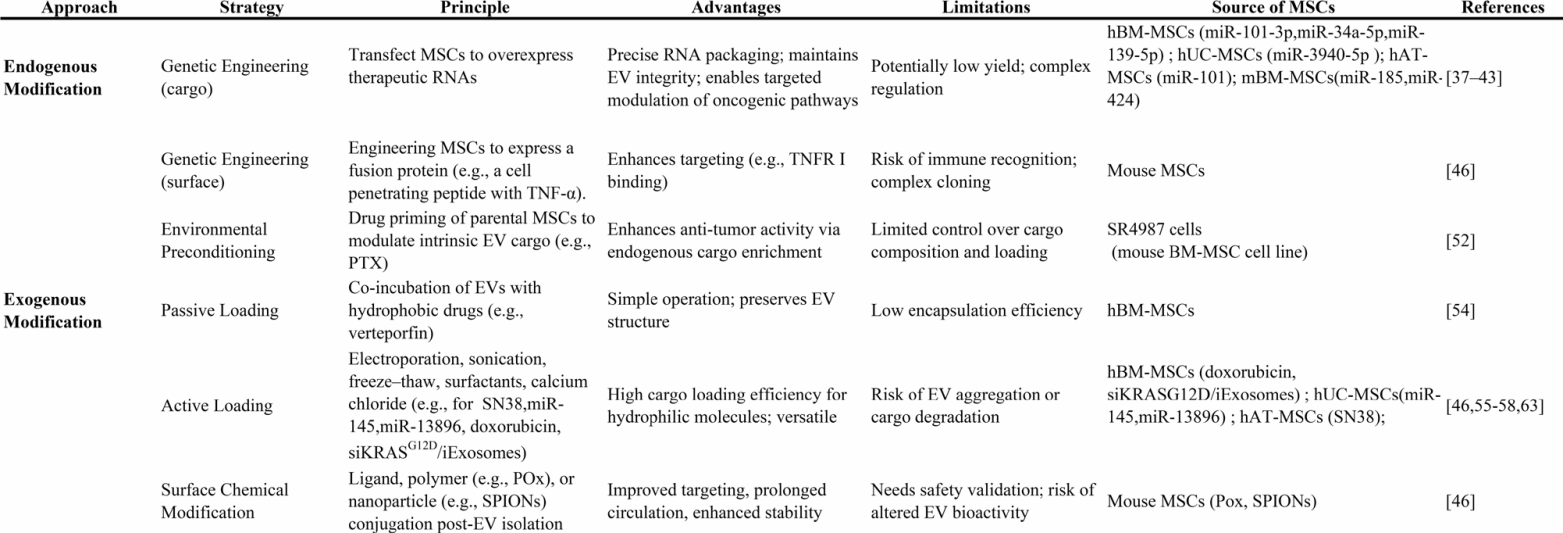
Strategies for engineering MSC-EVs for drug loading and functional modification

### Endogenous modification of MSC-EVs

Endogenous modification was achieved by directly modulating MSC parent cells before EV isolation, which can be achieved by genetic or biochemical engineering of parent MSCs to overexpress therapeutic RNAs or proteins and environmental preconditioning stimulation to modulate EV contents. Despite some unpredictable “black-box” aspects, this approach remains a powerful tool in EV-based biotechnology.

#### Genetic engineering for noncoding RNA loading

The genetic engineering of MSCs to overexpress therapeutic RNAs, such as miRNAs/siRNAs, enables the production of EVs enriched with nucleic acids, subsequently facilitating the targeted delivery of regulatory nucleic acids to tumor cells. This approach allows precise modulation of oncogenic signaling pathways in recipient cells, offering a promising strategy for cancer therapy. It is important to note that synthetic miRNA mimetics by themselves typically lack the natural sorting sequences that guide them into EVs. As a result, simply increasing the intracellular concentration of the desired miRNA through transfection or lentiviral transduction leads to its passive incorporation into the secreted EVs during biogenesis.

For example, in HNSCC, human bone marrow-derived MSCs (hBM-MSCs) transfected with miR-101-3p mimics produced EVs enriched with miR-101-3p, which effectively suppressed tumor progression by downregulating Collagen Type X Alpha 1(COL10A1) [37]. In a 7,12**-**dimethylbenzanthracene (DMBA)-induced oral potentially malignant disorder (OPMD) model, EVs derived from mouse bone marrow-derived mesenchymal stem cells (mBM-MSCs) transfected with a miR-185 mimic exhibited anti-tumorigenic effects by attenuating inflammation, reducing epithelial dysplasia, inhibiting proliferation and angiogenesis, and promoting apoptosis [38]. In CRC, MSC-derived EVs containing miR-34a-5p, which were generated through transfection of the hBM-MSCs with miR-34a-5p mimics, modulated the MYC proto-oncogene (c-MYC)/DNA methyltransferase 3a (DNMT3a)/ phosphatase and tensin homolog deleted on chromosome 10 (PTEN) axis to inhibit tumor growth and regulate epigenetic and proliferative pathways [39]. Similarly, in human umbilical cord-derived MSCs (hUC-MSCs), transfection with miR-3940-5p mimics led to EVs that suppressed EMT, invasion, metastasis, and tumor progression by targeting integrin subunit alpha 6 (ITGA6) and inhibiting the transforming growth factor beta 1(TGF-β1) pathway [40].

In the study of osteosarcoma, EVs produced by human adipose-derived mesenchymal stem cells (hAT-MSCs) were genetically modified via lentiviral transduction to overexpress miR-101. These miR-101-enriched EVs significantly reduce invasion and migration in vitro and inhibit metastatic progression in vivo without observable side effects, primarily through the downregulation of B-cell lymphoma 6 (BCL6) [41]. In ovarian cancer, mBM-MSCs transfected with miR-424 mimics produced EVs that downregulated MYB proto-oncogene (MYB) expression, thereby impairing tumorigenesis and angiogenesis [42]. In bladder cancer, lentiviral transduction of hBM-MSCs to express miR-139-5p resulted in EVs that targeted kinesin family member 3a (KIF3A), activated cyclin-dependent kinase inhibitor 1 (p21), and suppressed tumor progression [43]. These examples highlight the promise of MSC-EVs as programmable, cell-free vehicles for noncoding RNA-based cancer therapies, offering targeted gene modulation with reduced systemic toxicity.

#### Genetic engineering of MSC-EV surface proteins

In addition to genetic loading of the EV cargo, surface functionalization via membrane protein engineering has become a widely adopted strategy to improve targeted delivery. While MSC-EVs naturally exhibit tumor-homing capabilities, surface protein modification can further improve the precision of delivery and enable the presentation of therapeutic molecules [44–46]. A key principle in this field is fusing targeting ligands or peptides with EV-enriched transmembrane proteins such as lysosomal-associated membrane protein 2b (Lamp2b) and tetraspanins (CD9, CD63, and CD81). This approach has been successfully demonstrated in various EV-producing cells, providing a foundational strategy that can be effectively extended to the engineering of MSC-EVs. For example, the fusion of the rabies virus glycoprotein (RVG) peptide with Lamp2b enables mouse microglial-derived EVs to specifically target neurons by binding to nicotinic acetylcholine receptors. This strategy holds significant promise for the delivery of therapeutics across the blood‒brain barrier, suggesting potential applications in the treatment of brain tumors [47–49]. Similarly, Lamp2b fused with interleukin-3 (IL-3) has been used to direct HEK293T-derived EVs to leukemic cells that express the IL-3 receptor [50]. Another notable example, Kanuma et al., demonstrated that the conjugation of the ovalbumin (OVA) antigen to CD63 (CD63-OVA) significantly enhanced OVA on the surface of murine NIH3T3-derived EVs (OVA-EVs). These engineered OVA-EVs function as effective cancer vaccines, eliciting strong OVA-specific CD4⁺ and CD8⁺ T-cell responses and promoting the regression of xenograft tumors [51]. These examples establish a versatile and robust platform for further MSC-EVs therapeutic applications.

In addition to surface targeting, genetic modification of MSC-EV membranes can also serve to anchor therapeutic molecules. For example, Zhuang et al. engineered mouse MSCs to express a fusion protein comprising a cell penetrating peptide (CPP) and tumor necrosis factor-alpha (TNF-α), resulting in the production of exosomes with membrane-bound TNF-α (CTNF-α-exosomes). These CTNF-α-exosomes exhibited enhanced binding to tumor necrosis factor receptor I (TNFR I), thereby promoting TNF-α-mediated apoptosis in cancer cells [46]. This direct application of MSCs highlights their unique potential as a cellular source for producing therapeutically potent EVs.

#### Environmental preconditioning

In addition to genetic strategies, environmental preconditioning has also been leveraged to increase the therapeutic loading capacity of EVs. Pascucci et al. reported that MSC-EVs can successfully incorporate paclitaxel (PTX) following MSC priming with the drug, thereby supporting the potential of MSC-EVs as a novel vehicle for chemotherapeutic drug delivery [52]. These types of environmental preconditioning offer several practical advantages, including operational simplicity, cost-effectiveness, and scalability for large-scale EV production, without compromising structural integrity or functional potency. However, this approach also presents certain limitations. The lack of precise control over cargo loading may limit the reproducibility and predictability of therapeutic outcomes, particularly in cancer treatment.

### Exogenous modification of MSC-EVs

Exogenous modification, which includes cargo loading and surface modification, is conducted by direct modification of isolated EVs. Strategies for encapsulating drugs, RNA, into isolated MSC-EVs can be broadly classified into passive loading and active loading. Passive loading that applies primarily to hydrophobic drugs. Drugs are coincubated with EVs in a hydrophilic environment and passively dissolve into the phospholipid bilayer of EVs through self-assembly, which is simple to perform and does not harm the EVs, accompanied by a lower drug encapsulation efficiency than that of active methods; however, this can be addressed by increasing the drug concentration [53]. For example, microfluidic platforms have been developed to efficiently load hydrophobic drugs such as verteporfin (VP), a porphyrin compound with therapeutic potential against neuroblastoma, into MSC-EVs. By leveraging precise on-chip mixing and tunable incubation, these systems achieve superior loading efficiency while maintaining EV integrity and function, outperforming conventional benchtop methods and avoiding the damaging conditions often observed in other microfluidic techniques [54].

Active loading strategies transiently disrupt EV membranes to facilitate the entry of hydrophilic cargoes, such as nucleic acids. Common methods include electroporation, low-frequency ultrasound, freeze‒thaw cycles, and surfactant treatment, each of which enables effective drug encapsulation through increased membrane permeability. For example, the freeze‒thaw method has been demonstrated to significantly increase drug encapsulation efficiency in MSC-derived EVs. When combined with incubation and surfactant treatment, this technique allowed the successful loading of the hydrophobic drug 7-ethyl-10-hydroxycamptothecin (SN38) into MSC-EVs, achieving an encapsulation efficiency of 58% [55]. EVs derived from hUC-MSCs, enriched with miR-13896 by electroporation, were shown to specifically target tumor sites and suppress gastric cancer progression by downregulating the autophagy-related gene Autophagy-related protein 2 homolog A (ATG2A), thus inhibiting tumor cell proliferation and metastasis both in vitro and in vivo [56]. In CRC, miR-145 loaded onto EVs derived from hUC-MSCs suppresses tumor progression by downregulating fascin actin-bundling protein 1(FSCN1) expression. Efficient loading of miR-145 into EVs was achieved via a synergistic approach that combines sonication and electroporation [57]. In particular, Mukhopadhya et al. loaded the anticancer chemotherapeutic drug doxorubicin (Dox) into MSC-EVs by passive loading or active loading via either electroporation or sonication. The results indicated that electroporation appears to be superior for achieving maximum drug loading while not damaging surface proteins [58, 59]. Currently, electroporation is the most widely employed method for loading exogenous cargo into EVs [60–62].

Additional chemical modifications can also be applied to the surface of MSC-EVs after isolation to further enhance their tumor-targeting capabilities. Zhuang et al. coupled these CTNF-α-exosomes with superparamagnetic iron oxide nanoparticles (SPIONs), resulting in CTNF-α-exosome-SPIONs. In vivo studies demonstrated that TNF-α-loaded exosome-based vehicle delivery enhanced cancer targeting under an external magnetic field, leading to enhanced antitumor efficacy and reduced systemic toxicity [46]. Simon et al. demonstrated that the modification of MSC-derived EVs with polyoxazolines (POx) bearing lipid anchors effectively stabilizes EVs in plasma and modulates their biodistribution while preserving their native characteristics. Compared with both unmodified EVs and polyethylene glycol (PEG) modified EVs, which constitute the current “gold standard” surface modification strategy, POxylation not only maintains but also appears to enhance the immunomodulatory properties of MSC-EVs. These findings highlight POxylation as a promising alternative approach for optimizing the pharmacokinetic profile and therapeutic potential of EV-based nanotherapeutics [44].

## Current challenges in MSC-EVs-based cancer therapies

The ongoing Phase I clinical trial, NCT03608631, is currently the only clinical trial applying MSC-EVs for cancer treatment that is registered on www.clinicaltrials.gov. This landmark trial is investigating the safety, tolerability, and optimal dosing of engineered MSC-EVs, known as iExosomes, which are exogenously loaded with siRNAs targeting the KRAS^G12D^ mutation in patients with metastatic pancreatic cancer. KRAS^G12D^ is a key oncogenic driver in pancreatic ductal adenocarcinoma (PDAC), playing a central role in both tumor initiation and progression. In this trial, iExosomes are systemically administered to exploit their natural tumor-tropic behavior and their ability to deliver therapeutic RNA molecules directly to tumor cells. Preclinical studies have shown that MSC-EVs can effectively transport siRNA to KRAS^G12D^ -mutant cells, leading to significant suppression of oncogenic signaling pathways and tumor regression. The treatment also led to increased intratumoral infiltration of CD8 + T cells, suggesting a role in immunomodulation [63]. The results of this unique trial highlight the potential and promise of MSC-EVs as a programmable, cell-free platform for noncoding RNA-based cancer therapies, offering targeted gene modulation with reduced systemic toxicity.

Despite the promising therapeutic potential of MSC-EVs, their clinical translation remains hindered by key challenges, such as the source of the MSCs for producing EVs, low production yield, inefficient cargo loading, and batch-to-batch variability. MSC-EVs have demonstrated a dual role, exhibiting both pro-tumor and anti-tumor functions, similar to MSCs. This contradictory behavior is largely attributed to the heterogeneity of MSCs and the substances they carry. A key factor influencing this dual function is the tissue source of the MSCs. Studies have shown that MSCs-EVs derived from different tissues have distinct effects on tumors. For example, EVs secreted by hBM-MSCs have been observed to promote the proliferation, migration, and invasion of osteosarcoma cells by transferring miR-208a [64]. Similarly, EVs from hAT-MSCs secrete exosomes containing platelet-derived growth factor, which promotes tumor progression by encouraging angiogenesis [65]. In contrast, EVs from hUC-MSCs have been shown to retard the progression of esophageal squamous cell carcinoma by carrying miR-375, which downregulates Enabled homolog (ENAH) [66]. A meta-analysis highlighted this difference, indicating that only 26% of studies reported an onco-suppressive effect for hBM-MSCs and 46% for hAT-MSCs, whereas 88% confirmed a tumor-suppressive role for hUC-MSCs [67]. This underscores that the origin of MSCs dictates the molecular cargo of the EVs, thereby determining their functional outcome in the tumor microenvironment. Given their higher tumor-suppressive potential, hUC-MSCs-EVs are considered a superior choice for developing EV-based cancer treatments.

Furthermore, the specific conditions of the TME itself, such as hypoxia or the presence of inflammatory cytokines, can alter the cargo of the MSC-EVs, shifting their function from anti-tumor to pro-tumor [36, 68–71]. For example, hypoxia-induced enrichment of let-7f in MSC-EVs has demonstrated antitumor effects in breast cancer models [72]. However, Lo Sicco et al. reported that MSC-EV treatment could induce M2 macrophage polarization, which was further amplified by hypoxic preconditioning of MSCs [73]. While M2 macrophages play beneficial roles in tissue repair and regeneration [74], their immunosuppressive and tumor-promoting functions within the TME render them detrimental in the context of cancer therapy [75, 76]. Hypoxia has also been shown to augment the anti-inflammatory properties of MSC-EVs, thereby improving their ability to modulate immune responses and reduce inflammation [77]. Similarly, preconditioning MSCs with inflammatory stimuli such as TNF-α, Lipopolysaccharide (LPS), or Interferon-gamma (IFN-γ) enhances the immunomodulatory capacity of their EVs, in part by increasing the levels of miRNAs such as miR-181 and promoting M2 macrophage polarization [78–80]. These findings underscore the critical risk associated with exposing MSCs directly to the TME, as it may induce the secretion of pro-tumorigenic EVs. To mitigate these risks, a more controlled and safer approach involves the ex vivo engineering of MSC-EVs, particularly those derived from hUC-MSCs, with defined therapeutic payloads such as tumor-suppressive siRNAs or miRNAs. In addition, modifying EVs to express tumor-targeting ligands can enable selective delivery to cancer cells, thereby maximizing therapeutic efficacy while minimizing off-target effects. These strategies not only enhance the therapeutic potential of MSC-EVs but also eliminate the risks associated with direct MSC administration, which may lead to pro-tumorigenic reprogramming under the influence of the TME.

Other challenges include low production yield, inefficient cargo loading, and batch-to-batch variability. To address this challenge, Pan et al. recently introduced nanochannel electroporation (NEP) as a superior alternative to traditional bulk electroporation (BEP). NEP offers precise and uniform cargo delivery while preserving EV integrity and enhancing scalability [81]. Moreover, prolonged in vitro expansion of MSCs can induce senescence, which may alter EV composition and therapeutic efficacy [82–84]. To minimize this variability, most clinical protocols use MSCs from earlier passages (e.g., passages 3 to 6) for cell therapy. Therefore, for MSC-EVs, strategies such as metabolic reprogramming and the use of early-passage cells should be incorporated into quality control pipelines to ensure consistency and reproducibility.

## Conclusions

Engineered MSC-EVs represent a promising and innovative platform for next-generation, cell-free cancer therapies. This review has highlighted recent advances in bioengineering approaches that enhance the therapeutic potential of MSC-EVs, including improvements in cargo loading efficiency, target specificity, and overall efficacy via endogenous and exogenous modifications. Despite their potential, the clinical translation of MSC-EVs faces several critical challenges. One major concern is their dual functional role in tumor biology, exhibiting both pro-tumorigenic and anti-tumorigenic effects depending largely on the tissue origin of the MSCs and the TME. Among various MSC sources, EVs from hUC-MSCs have demonstrated the most consistent tumor-suppressive activity, making them a preferred choice for clinical applications. Furthermore, to mitigate the risks of pro-tumorigenic effects and enhance therapeutic specificity, precise engineering approaches, such as loading tumor-suppressive miRNAs or siRNAs and incorporating targeting ligands are essential.

Additionally, product quality and therapeutic consistency, it is imperative to establish validated, good manufacturing practice (GMP)-compliant protocols for EV isolation, characterization, and storage. The integration of advanced engineering tools, such as microfluidic loading systems [54, 85], Artificial Intelligence (AI)-assisted EV profiling [86], and ligand-guided targeting strategies, will be crucial for optimizing therapeutic performance and enabling personalized cancer treatment. With continued innovation and clinical validation, MSC-EVs have the potential to become a transformative modality in precision oncology.

## Supplementary Information

Below is the link to the electronic supplementary material.

Supplementary Material 1.

Supplementary Material 2.

## Data Availability

No datasets were generated or analysed during the current study.

## Abbreviations

MSCs: Mesenchymal stem/stromal cells
MSC-EVs: Mesenchymal stem cell-derived extracellular vesicles
EVs: Extracellular vesicles
siRNAs: Small interfering RNAs
miRNAs: MicroRNAs
hUC-MSCs: Human umbilical cord-derived mesenchymal stem cells
GMP: Good Manufacturing Practices
TME: Tumor microenvironment
CRC: Colorectal cancer
HNSCC: Head and neck squamous cell carcinoma
EMT: Epithelial‒mesenchymal transition
DNA: Deoxyribonucleic acid
RNA: Ribonucleic acid
mRNA: Messenger RNA
MISEV2018: Minimal information for studies of extracellular vesicles 2018
ISEV: International Society for Extracellular Vesicles
MISEV2023: Minimal information for studies of extracellular vesicles 2023
ApoBDs: Apoptotic bodies
sEVs: Small extracellular vesicles
m/lEVs: Medium/large extracellular vesicles
ACDVs: Artificial cell-derived vesicles
SVs: Synthetic vesicles
LNP: Lipid nanoparticle
Endo-EVs: Endocytosis-derived extracellular vesicles
hnRNPA2B1: Heterogeneous nuclear ribonucleoprotein A2/B1
AGO2: Argonaute-2
Alix: ALG-2-interacting protein X
hBM-MSCs: Human bone marrow-derived mesenchymal stem cells
COL10A1: Collagen Type X Alpha 1
DMBA: 7,12-dimethylbenzanthracene
OPMD: Oral potentially malignant disorder
mBM-MSCs: Mouse bone marrow-derived mesenchymal stem cells
c-MYC: MYC proto-oncogene
DNMT3a: DNA methyltransferase 3a
PTEN: Phosphatase and tensin homolog deleted on chromosome 10
hAT-MSCs: Human adipose tissue-derived mesenchymal stem cells
BCL6: B-cell lymphoma 6
MYB: MYB proto-oncogene
KIF3A: Kinesin family member 3a
P21: Cyclin-dependent kinase inhibitor 1
Lamp2b: Lysosome-associated membrane glycoprotein 2b
RVG: Rabies virus glycoprotein
IL-3: Interleukin-3
OVA: Ovalbumin
CPP: Cell-penetrating peptide
TNF-α: Tumor necrosis factor-alpha
TNFR I: Tumor necrosis factor receptor I
PTX: Paclitaxel
VP: Verteporfin
SN38: 7-ethyl-10-hydroxycamptothecin
ATG2A: Autophagy-related protein 2 homolog A
FSCN1: Fascin actin-bundling protein 1
Dox: Doxorubicin
SPION: Superparamagnetic iron oxide nanoparticle
POx: Polyoxazoline
PEG: Plyethylene glycol
PDAC: Pancreatic ductal adenocarcinoma
ENAH: Enabled homolog
LPS: Lipopolysaccharide
IFN-γ: Interferon-gamma
NEP: Nanochannel electroporation
BEP: Bulk electroporation
AI: Artificial Intelligence
