# Supplementary material for: Harnessing engineered mesenchymal stem cell-derived extracellular vesicles for innovative cancer treatments
Source: Stem Cell Res Ther. 2025 Nov 18;16:648. doi: 10.1186/s13287-025-04708-5 (PMC12625093; doi:10.1186/s13287-025-04708-5)
Supplement: Supplementary file 2 — Supplementary Material 2. [file 13287_2025_4708_MOESM2_ESM.pdf]

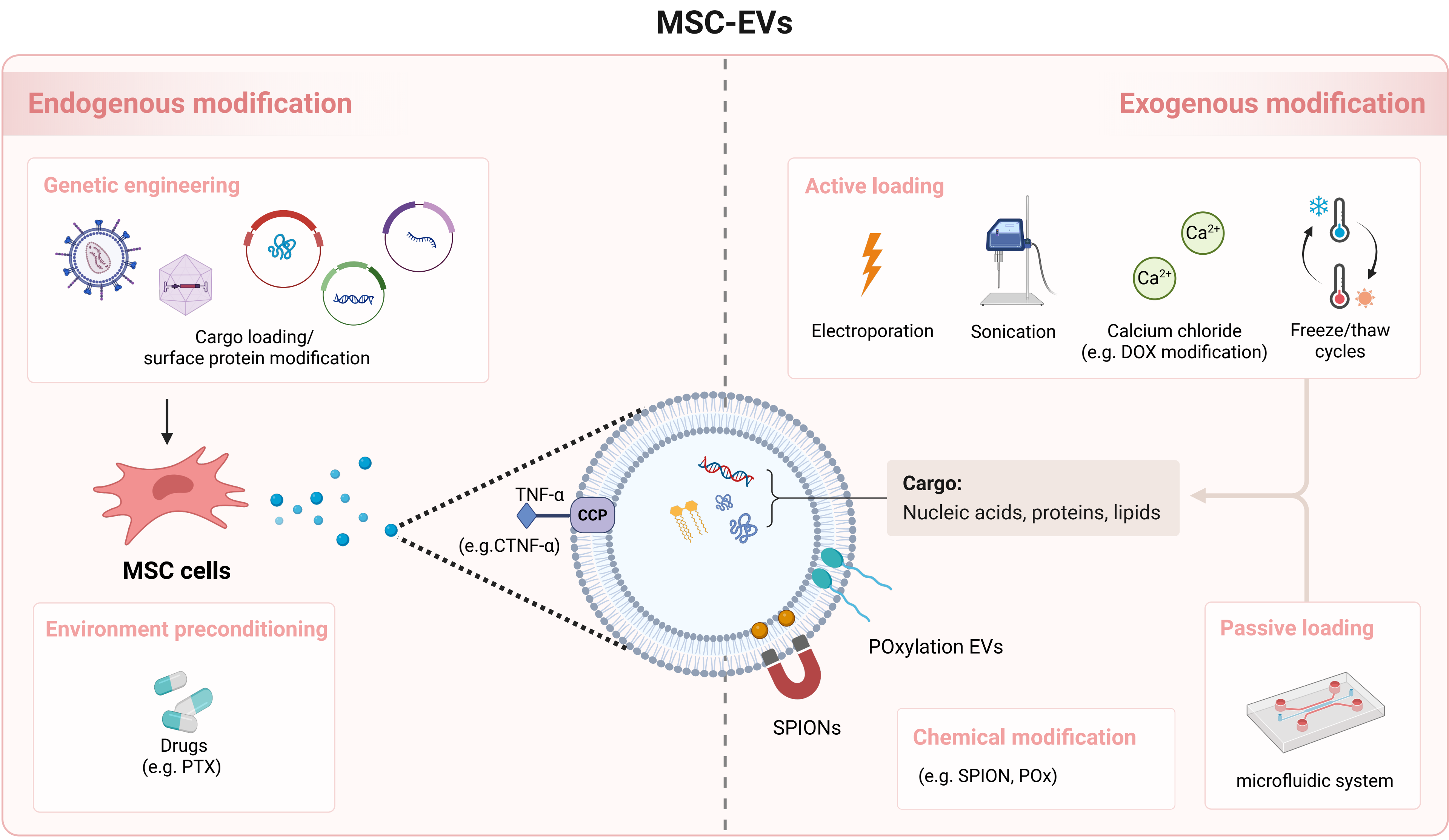
MSC-EVs
Endogenous modification
Exogenous modification
Genetic engineering
Cargo loading/
surface protein modification
Active loading
Ca2+
Ca2+
Electroporation
Sonication
Calcium chloride
(e.g. DOX modification)
Freeze/thaw
cycles
MSC cells
TNF-α
CCP
(e.g.CTNF-α)
Cargo:
Nucleic acids, proteins, lipids
POxylation EVs
SPIONs
Environment preconditioning
Drugs
(e.g. PTX)
Chemical modification
(e.g. SPION, POx)
Passive loading
microfluidic system
